# Evaluation of a Virtual Home Health Heart Failure Program: Mixed Methods Study

**DOI:** 10.2196/64877

**Published:** 2025-07-23

**Authors:** Nilufeur McKay, Rosemary Saunders, Helene Metcalfe, Sue Robinson, Peter Palamara, Kellie Steer, Jeannie Yoo, Miles Ranogajec, Lisa Whitehead, Beverley Ewens

**Affiliations:** 1 School of Nursing and Midwifery, Edith Cowan University, 270 Joondalup Drive, Joondalup, 6027, Australia, 61 863046116; 2 Ramsay Connect, Perth, Australia; 3 Australian Unity, Sydney, Australia; 4 College of Medicine and Public Health, Flinders University, Bedford Park, Australia; 5 Ramsay Connect, Geelong, Australia

**Keywords:** heart failure, patient care team, telemedicine, virtual health, mixed methods study, healthcare systems, supportive care, virtual healthcare, monitoring support program, quality of life, Australian, value-based healthcare

## Abstract

**Background:**

Heart failure is a prevalent and debilitating condition, affecting millions globally and imposing a significant burden on patients, families, and health care systems. Despite advancements in medical treatments, the gap in effective, continuous, and personalized supportive care remains glaringly evident. To address this pressing issue, virtual health care services delivered by interdisciplinary teams represent a promising solution. Understanding the outcomes and experience of remote monitoring–enabled interdisciplinary chronic disease management programs can inform resource allocation and health care policy decisions.

**Objective:**

The purpose of this study was to evaluate the clinical and behavioral outcomes of patients undertaking a Virtual Home Health Heart Failure Program (VHHHFP) and explore the experiences of patients and health care practitioners (HCPs).

**Methods:**

The VHHHFP is a virtual postdischarge support service for patients with heart failure that includes an intensive 3-month period followed by a maintenance period delivered by an interdisciplinary team. A mixed methods study was conducted with patients and HCPs. Self-reported outcome data (KCCQ-12 [Kansas City Cardiomyopathy Questionnaire-12], PHQ-4 [Patient Health Questionnaire-4], PAM-13 [Patient Activation Measure-13], and PREMs [Patient Reported Experience Measures]) were obtained from the records of patients (N=49) who completed the intensive phase of the VHHHFP, and interviews were conducted with patients (n=9) and HCPs (n=6). A paired *t* test was used to compare quantitative data before and after the 3-month intervention, and a thematic qualitative analysis was undertaken of interview data.

**Results:**

Thirty-one of the 55 (77.5%) patients completed the baseline and 3-month follow-up KCCQ-12 assessment. The mean KCCQ-12 summary score at 3 months was 72.20 (SD 20.2), which was significantly higher than the mean summary score at baseline of 50.51 (SD 17.59; *P*<.001). These findings were similar for the KCCCQ-12 subscales: physical limitations (mean 47.09, SD 29.7 and mean 69.43, SD 22.6; *P*<.001), quality of life (mean 43.75, SD 21.7 and mean 62.91, SD 25.7; *P*<.001), symptom frequency (median 60.40, IQR 1-100 and median 91.70, IQR 35.40; *P*<.001), and social limitation (median 50.0, IQR 1-100 and median 82.50, IQR 32.50; *P*<.001). The PHQ-4 measure of psychological health was completed by 32 (80%) patients. The median scores at baseline and follow-up for total distress (median 1.50, IQR 0-7 and median 0.0, IQR 0-8; *P*<.02), and the anxiety subscale (median 1.0, IQR 0-6 and median 0.0, IQR 0-4; *P*<.02) reduced over time. Six hospital admissions were recorded (10.2% of 49 patients) within 30 days. Nine patient interviews aligned with the value-based health care (VBHC) Capability, Comfort, and Calm (CCC) framework. Three themes were identified, which are as follows: (1) enhanced patient capability, (2) improved patient comfort, and (3) positive influences on calm. Six health care professionals shared experiences of the VHHHFP, with three emerging themes: (1) improved patient capability through shared decision-making, (2) improving capability through care practices, and (3) promoting comfort and calm through virtual coordination and collaboration.

**Conclusions:**

The use of technologies to support the management of HF is an area of growth. This study contributes to the understanding of how remote patient monitoring with interdisciplinary chronic disease support, integrated into an existing system, can improve clinical outcomes for patients.

## Introduction

Heart failure (HF) has been acknowledged as one of the Western world’s most significant public health issues [1]. This chronic condition results in reduced quality of life, creating a burden for health care systems in terms of resource use and financial cost [2-4]. Globally, HF is described as an epidemic, affecting more than 64 million people worldwide [5], and a diagnosis of HF is associated with high rates of morbidity and mortality, particularly in low- and middle-income countries [6]. The percentage of the population diagnosed with HF around the world varies among populations [7], but globally is estimated to be between 1% and 3% of the total population [8]. In Australia, it is estimated that 1%‐2% of the population are diagnosed with HF, compared to a prevalence of 2.4%‐3% in the United States [2] and between 1.3% and 6.7% in Asia [3]. In 2017‐2018, an estimated 102,000 (0.5%) people self-reported living with HF within Australia, with around 179,000 hospitalizations in 2020‐2021 attributed to HF or cardiomyopathy as the primary diagnosis [9]. The prevalence of HF is predicted to increase due to the aging population, improved treatment of acute cardiac events, and availability of evidence-based therapies for those with HF [2]. It is estimated that by 2023, cases of HF in Australia will increase to 750,000 [10]. The majority of health care costs for people with HF are associated with an increasing rate of hospitalizations due to poor self-care, nonadherence to treatment, or inability to access medications [4]. Research suggests that most patients (80%) living with HF are reliant on their general practitioner (GP) for ongoing management and support [11].

A growing body of evidence supports the use of digital health technology in improving patient outcomes [12-14], with telemonitoring [15] and digital health becoming central to health care [16]. Virtual health care has become an indispensable component of contemporary care delivery, which enables those with chronic conditions to stay connected to online supportive environments and clinicians to establish two-way communication and noninvasive monitoring for patients in remote locations [17]. The COVID-19 pandemic expedited the adoption of telehealth globally. However, the evaluation of telehealth outcomes has not necessarily matched the pace of its uptake [18]. In response, there has been an increase in the exploration of remote and virtual patient monitoring and care models to manage and improve the outcomes of patients with HF [12]. However, the use of virtual HF programs remains in its infancy in Australia. A recent systematic review identified that telemonitoring, remote patient management, and patient self-empowerment as an integrated approach performed best in terms of readmission rates and overall hospital visits [17], with further research into this approach needed [19]. This study aimed to evaluate the clinical and behavioral outcomes of patients undertaking a Virtual Home Health Heart Failure Program (VHHHFP) and to explore the experiences of patients and health care practitioners (HCPs) who participated in the program.

## Methods

### Research Design and Study Population

A mixed methods study was conducted in collaboration with patients and HCPs. Self-reported outcome data (KCCQ-12 [Kansas City Cardiomyopathy Questionnaire-12], PHQ-4 [Patient Health Questionnaire-4], PAM-13 [Patient Activation Measure-13], and PREMs [Patient Reported Experience Measures]) were obtained from the records of 55 patients who completed the intensive phase (0‐3 months) of the VHHHFP. Interviews were conducted with 9 patients and 6 HCPs. A mixed methods approach was selected as it provides the opportunity to integrate quantitative findings in the analysis of the qualitative data [20]. This is particularly important in the evaluation of a chronic disease management program, where there is a need to understand from a patient’s perspective how programs impact or fail to impact health outcomes observed from a purely quantitative approach. This study method provides a richer level of understanding of content, processes, and policies within programs [21]. The study is reported in accordance with the mixed methods reporting guidelines by Lee et al [22]. The study population comprised patients participating in the intensive phase of the program, the clinicians delivering the program, and other clinicians external to the program but involved in the care of the patients.

### The VHHHFP

The VHHHFP is a virtually delivered postdischarge support service for patients with HF. The program aims to (1) improve HF symptoms, quality of life, and physical and social limitations, (2) improve HF self-management skills and capabilities, (3) improve patient understanding of medications and therapy adherence, (4) reduce signs and symptoms of anxiety and depression associated with HF, and (5) reduce preventable hospital admissions through collaborative care practices.

**Figure 1.**
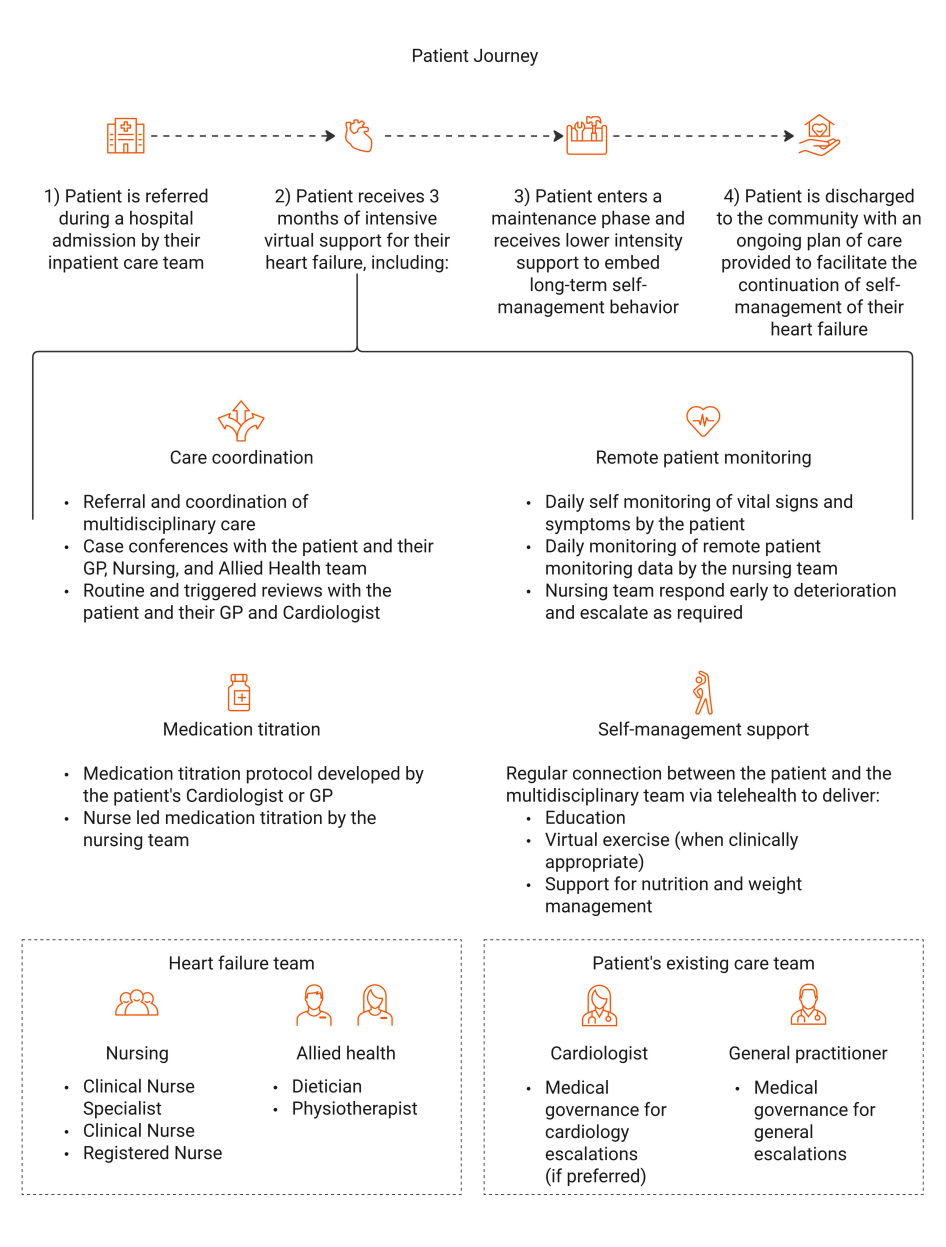
The patient journey through the Virtual Home Health Heart Failure Program (a 3-months intensive phase followed by a maintenance period). GP: general practitioner.

Suitable patients who meet the inclusion criteria (Textbox 1) can be referred to the program by a member of their inpatient care team during a hospital admission. Services are delivered via telehealth by a clinical nurse specialist (CNS), a clinical nurse or registered nurse, a dietitian, and a physiotherapist. Interventions provided include care coordination, remote patient monitoring of vital signs and symptoms, nurse-led medication titration (as directed by the patient’s cardiologist or GP), education, virtually delivered exercise (when clinically appropriate), and support for nutrition and weight management. The necessary equipment is provided to patients at no cost. The program operates during business hours, with an HF action plan provided to patients for out-of-hours concerns. The program integrates with primary and specialist care teams, with medical governance either provided by the patient’s existing cardiologist or GP, as per the specialist’s preference. The program’s intensive phase is delivered over a period of 3 months and includes an initial visit by one of the VHHHFP clinical nurses, who described the program to the patient and obtained consent to participate in the program. Initial screening surveys were completed, and the patient received a comprehensive description of the home monitoring equipment. Upon discharge, the patient received intensive care coordination by an interdisciplinary team with remote monitoring, medication titration, and self-care management support. More details of these individual components are represented in Figure 1. Following this, the patient enters a 3‐12-month maintenance phase based on the patient’s needs to embed long-term self-management behaviors. The maintenance phase of the program was not included in this study. Upon completion, the patient is discharged to their primary care clinician with an ongoing plan of care for their HF. At this point, the patient does not have further access to the VHHHFP.

Several validated self-reporting assessment tools were used to monitor the patient’s progress while on the program. The measures were selected to be consistent with the health outcome measures for patients with HF recommended by the International Consortium for Health Outcomes [23]. Self-reported outcomes included the KCCQ-12 [24], the PHQ-4 for anxiety and depression [25], and the PAM-13 [26] for assessing engagement with health care and self-management, and a PREM survey. The PREM survey was adapted and modified from the Australian Hospital Patient Experience Question Set [27] to suit the context of the VHHHFP.

Textbox 1. **Virtual Home Health Heart Failure Program inclusion and exclusion criteria.**

**Patient inclusion criteria**

- New or existing diagnosis of heart failure (heart failure with reduced ejection fraction (HFrEF) or heart failure with preserved ejection fraction (HFpEF).
- New York Heart Association (NYHA) functional class I, II, or III.
- Patient referral to the Virtual Home Health Heart Failure Program (VHHHFP) from a clinician at one of the three hospital sites.

**Exclusion criteria**

- Discharged to a residential aged care facility.
- NYHA functional class IV.
- Hemodynamic instability.
- Recurrent arrhythmias or unstable angina requiring investigation.
- Deteriorating renal function, defined as estimated glomerular filtration rate (eGFR) <30 mL/min.
- Continuous oxygen requirement at rest.
- Existing cognitive impairment.

### Recruitment and Consent

#### Patient Participants

Patients provided consent for their quantitative data to be collected for evaluation on commencement of the program. Patients enrolled in the intensive phase of the VHHHFP were sent an email by the HF CNS, informing them about the study and the process to participate in an interview. Patients who were interested in participating in the semistructured interview contacted the study research assistant via email and were given the opportunity to ask any questions they had. Written informed consent was obtained before the commencement of the interviews.

#### Health Care Practitioner Participants

HCPs (including HF CNSs, physiotherapists, GPs, and cardiologists) were eligible to participate if they had delivered care to the patients within the 3-month intensive phase of the program. Health care participants involved in the care of the recruited patient participants were sent an email to inform them about the study, and who then contacted the research team directly. HCP participants provided written consent before the commencement of interviews.

#### Setting

Three hospital sites across Australia, which varied in size from 250 to 900 beds.

#### Data Collection

Data were collected via surveys and individual semistructured interviews. The quantitative data were collected as part of the VHHHFP, and the qualitative data were collected to explore patients’ and stakeholders’ perspectives of the program.

#### Quantitative Data Collection

Quantitative data included baseline demographic information and patient self-reported outcome data via the home digital platform as part of the VHHHFP. Baseline patient demographic and clinical characteristics collected included age, identified gender, HF subtype (heart failure with reduced ejection fraction or heart failure with preserved ejection fraction), NYHA Functional Class, and the Length of stay, Acuity on admission, Comorbidity, and Emergency room usage (LACE) index as a measure of patients’ risk of 30-day readmission [28]. This information was extracted by a program administrator and provided to the research team. Patients completed the self-reported outcomes directly on the home digital platform at various times during the intensive phase. Pretest data (baseline) and posttest data (3 months after enrollment) were collected. Patients were followed up at key time points, including enrollment (baseline) and at 12 weeks, to measure health status (KCCQ-12), PREMs, anxiety and depression (PHQ-4), and engagement with health care and self-management (PAM-13). Data submitted up to 14 days before or post the expected completion date (70‐98 d) of the 12-week program were included for analysis. A secure messaging platform was used to transfer patient data to the research team. Data did not contain identifiable patient data and were grouped via an ID number for analysis on a per-patient basis. For those participants who took part in the qualitative phase of the study, IDs were linkable via a separately stored key that provided patient contact details.

#### Qualitative Data Collection

Individual interviews were conducted to obtain information about the patient and clinician perspectives of the program. The value-based health care (VBHC) Capability, Comfort, and Calm (CCC) framework [29] guided the development of the patient and HCP interview questions (Multimedia Appendix 1). Patients were given a choice to complete interviews in person or virtually. The interviews were audio-recorded and later transcribed by a member of the research team. Patients were given the option to be interviewed individually or with a family member, and permission was sought to audio record the interview. One-to-one individual interviews with health care providers were undertaken by a member of the research team who did not have a professional relationship with the participants. Health care provider participants responded to 8 open-ended questions (Multimedia Appendix 1). Interviews were audio-recorded and were up to an hour in duration.

#### Quantitative Analysis

All data were exported from Microsoft CSV files to SPSS (version 28.0.1; IBM Corp) for analysis. Frequency counts were used to describe data pertaining to patient demographics and clinical status, program experience, and hospital readmissions. The normality of the data on patients’ physical and psychological health status and health care behaviors at each time point was assessed graphically and numerically using the Shapiro-Wilk Test for small sample sizes. For data that was normally distributed, the mean and SD were used as measures of central tendency and dispersion, and dependent-samples *t* tests were used to test for changes over time (Time 1 and Time 2) in patient-reported outcomes. For data that were nonnormally distributed, the median and IQR, and the Wilcoxon signed-rank test for dependent samples were used. Frequency counts were also provided for patient demographic and clinical status data. Patient datasets that had missing pre- or postsurvey data were excluded from the analysis. Patients who died during the study were excluded as required by the organization’s ethics committee.

#### Qualitative Analysis

Thematic analysis was then undertaken using Braun and Clarke’s six-step approach (familiarization with the data, generating codes, constructing themes, revising and defining themes, and producing the report) [30] to identify patterns of meaning to understand the patient and HCPs’ experiences of the VHHHFP. The audio recordings were transcribed verbatim and verified against the recording, and the transcripts were read several times before initial coding by one researcher (HM) and then independently by two researchers (RS and NM). Following initial coding, the researchers discussed the coding and reached consensus on categories. Data were organized and managed using Microsoft Excel. Research team discussions then informed the development of themes and subthemes using the VBHC framework [31], comprising the concepts of CCC.

To ensure qualitative rigor and trustworthiness, data were collected until data saturation was achieved, which was based on the appraisal of the data collected and the rich dialogue that related to the study aim. Member checking was not possible due to the single participant interaction. Discussion and interpretations of the data ensured credibility. The presentation of the findings will guide other researchers in the transferability of the findings. The researchers maintained a clear record of the reflexive analysis process, supporting the confirmability of the findings.

### Ethical Considerations

The research study was conducted following the Australian National Statement on Ethical Conduct of Human Research (2023), developed by the National Health and Medical Research Council, the Australian Research Council, and Universities Australia. Permission to conduct the study was sought and approved by an Australian institutional review board, the Ramsay Health Care Human Research Ethics Committee (approval no 2022/PID/2031), and the Edith Cowan University Human Research Ethics Committee (approval no 2022-‐03864). Research procedures were followed under the ethical standards of the approving national Human Research Ethics Committees and with the WMA Declaration of Helsinki. Patients provided informed consent for their quantitative data to be collected for evaluation at the commencement of the program. Patients enrolled in the intensive phase of the VHHHFP were invited to participate in an interview via an email from the HF CNS, which informed them about the study and the process to participate in an interview. Patients and staff interested in participating in the qualitative component of the study were required to contact the study research assistant via email. All participants were provided with a participant information form, which provided detailed information about the study and what their involvement comprised. Potential participants were also informed they would receive a US $35 gift voucher as acknowledgment of their semistructured interview participation. Written informed consent was obtained before the commencement of all interviews. Participants were informed that their participation was voluntary and that they were free to withdraw from the study at any time; however, following data analysis, their data could not be removed. No personal identifying information was collected.

## Results

### Baseline Characteristics

From June 1, 2022, to November 30, 2022, a total of 55 patients were enrolled into the VHHHFP across 3 Australian hospital sites. Data for 5 patients were not reported in line with the ethical approval granted: 3 patients died during the intensive phase of the program, and 2 died during the data collection period following completion of the intensive phase of the program. In addition, 1 patient who enrolled but did not complete any questionnaire or biometric information was excluded. The demographic and clinical characteristics of the remaining 49 patients who enrolled and engaged with the program are presented in Table 1. Of these 49 patients, 7 withdrew and did not complete the program: 3 patients due to hospital admission, and 2 patients failed to complete the intensive phase of the program within the nominated time (98 days from enrollment data). Data for these 7 patients related to changes in physical and psychological health and health care behavior, as well as their experiences with the program, were subsequently excluded from the analysis.

**Table 1.** Demographic and clinical characteristics of patients enrolled and engaged in the Virtual Home Health Heart Failure Program.

Variable	Patient, n (%)
Sex (N=49)	
Male	31 (63)
Female	18 (37)
Age (years; N=49)	
41‐50	3 (6)
51‐60	4 (8)
61‐70	17 (35)
71‐80	17 (35)
81‐90	8 (16)
NHYA^a^ classification (N=42)	
I	0 (0)
II	34 (81)
III	7 (17)
IV	1^b^ (2)
Ejection fraction (%; N=47)	
40 and below	38 (81)
41‐49	3 (6)
50‐70	6 (13)
LACE^c^ score (N=34)	
0‐4	2 (6)
5‐9	13 (38)
10 and above	19 (56)

a NHYA: New York Heart Association.

b Although patients’ initial evaluation was NYHA classification IV, this patient was included due to cardiologist recommendations.

c LACE: Length of stay, Acuity of admission, Comorbidity, and Emergency room usage.

#### Patients’ Physical and Psychological Health Status and Health Care Behaviors

Descriptive statistics for measures of physical and psychological health status and health care behaviors taken at the commencement of the program (baseline: within 0‐14 d of enrollment) and after the intensive phase (follow-up: within 70‐98 d of enrollment) are presented in Table 2. Thirty-one (77.5%) of the 40 patients with HF completed a first and follow-up KCCQ-12 assessment within the intensive phase of the VHHHFP. The mean summary score at follow-up was found to be significantly higher than the mean summary score at baseline. Consistent with this, the mean baseline and follow-up scores for the KCCCQ-12 subscale measures of physical limitations and quality of life were found to have significantly increased over time, as did the median subscale scores at baseline and follow-up for symptom frequency and social limitation.

**Table 2.** Descriptive statistics for patients’ physical and psychological health status and health care behaviors at baseline and follow-up.

Measure	Baseline period	Follow-up period	Statistic *t* (df) or *z* score	*P* value
KCCQ-12^a^, N=31				
Summary score	50.51 (SD 17.59)	72.20 (SD 20.28)	*t*=–5.91 (30)	<.001
Physical limitation	47.09 (SD 29.77)	69.43 (SD 22.62)	*t*=–4.02 (30)	<.001
Symptom frequency	60.40 (IQR 1-100)	91.70 (IQR 35.40)	*z*=–4.43	<.001
Social limitation	50.0 (IQR 1-100)	82.50 (IQR 32.50)	*z*=–4.88	<.001
Quality of life	43.75 (SD 21.71)	62.91 (SD 25.73)	*t*=–3.94 (29)	<.001
PHQ-4^b^, N=32				
Total distress	1.50 (IQR 0-7)	0.0 (IQR 0-8)	*z*=–2.42	<.02
Anxiety	1.0 (IQR 0-6)	0.0 (IQR 0-4)	*z*=–2.53	<.02
Depression	0.0 (IQR 0-4)	0.0 (IQR 0-4)	*z*=–1.39	=.16
PAM-13^**c**^, N=28	57.40 (SD 8.53)	68.26 (SD 12.38)	*t*=–4.81 (27)	<.001

a KCCQ-12: Kansas City Cardiomyopathy Questionnaire.

b PHQ-4: Patient Health Questionnaire-4.

c PAM-13: Patient Activation Measure-13.

The PHQ-4 measure of psychological health was completed by 32 (80%) of program patients within the intensive phase of the VHHHFP. The median scores at baseline and follow-up for total distress (1.50, IQR 0-7 and 0.0, IQR 0-8; *z*=–2.42; *P*<.02), and the anxiety subscale (1.0, IQR 0-6 and 0.0, IQR 0-4; *z*=–2.53; *P*<.02) significantly reduced over time, while no statistically significant change over time was found in the median subscale score for depression (0.0, IQR 0-4 and 0.0, IQR 0-4; *z*=−1.39; *P*=.16). A total of 28 (70%) program patients completed the PAM-13. Analysis of the mean baseline (57.40 SD 8.53) and follow-up (68.26, SD 12.38) scores for the PAM-13 showed a statistically significant increase in patients’ self-reported knowledge, beliefs, confidence, and skills about managing their HF throughout the intensive phase of the VHHHFP (*t*_27_=−4.81; *P*<.001).

#### Patient Reported Experience Measures

Of the 34 (85%) patients who responded to the PREMs questionnaire, all (100%) responded “always” or “mostly” to questions about their treatment and care (Table 3). Most patients (94%) responded that their views and concerns were always listened to. Twenty-six (76%) patients responded that they always knew how to recognize HF or heart attack symptoms and what to do next. Seven (21%) patients responded that they mostly knew how to recognize HF, and one responded that they only sometimes knew how to recognize HF symptoms. In relation to the patients’ experience of the technology of the VHHHFP, most patients were satisfied to varying degrees. Similarly, most patients (n=33, 97%) were satisfied to very satisfied that they had the knowledge required to use the technology.

**Table 3.** Frequency of patient-reported experience measures responses.

Scale and experience item	Response 1, n (%)	Response 2, n (%)	Response 3, n (%)	Response 4, n (%)	Response 5, n (%)	Total, n (%)
Response: 1=always, 2=mostly, 3=sometimes, 4=rarely, and 5=never						
My views and concerns were listened to.	32 (94)	0 (0)	1(3)	0 (0)	1 (3)	34 (100)
My individual needs were met.	30 (88)	0 (0)	4(12)	0 (0)	0 (0)	34 (100)
I felt cared for.	31 (91)	3 (9)	0 (0)	0 (0)	0 (0)	34 (100)
I was involved as much as I wanted in making decisions about my treatment and care.	30 (88)	4 (12)	0 (0)	0 (0)	0 (0)	34 (100)
I was kept informed as much as I wanted about my treatment and care.	33 (97)	1 (3)	0 (0)	0 (0)	0 (0)	34 (100)
The staff involved in my care communicated with each other about my treatment.	31 (91)	3 (9)	0 (0)	0 (0)	0 (0)	34 (100)
I knew how to recognize heart failure or heart attack symptoms, and what to do next if I experienced symptoms.	26 (76)	7 (21)	1 (3)	0 (0)	0 (0)	34 (100)
I felt confident in the safety of my treatment and care.	30 (88)	4 (12)	0 (0)	0 (0)	0 (0)	34 (100)
Response: 1=very satisfied, 2=satisfied, 3=neither, 4=dissatisfied, and 5=very dissatisfied						
How satisfied are you that the technology operated as expected? (ie, tablet, app, biometric devices, and video calls)	22 (65)	10 (30)	2 (6)	0 (0)	0 (0)	34 (100)
How satisfied are you that you have the knowledge to use the technology? (ie, tablet, app, biometric devices, and video calls)	26 (76)	7 (21)	1 (3)	0 (0)	0 (0)	34 (100)

#### Readmission to Hospital

The analysis of the patients’ admission to hospital during the intensive phase of the VHHHFP was undertaken on the 40 patients who completed the intensive phase of the VHHHFP within 70‐98 days and those patients who withdrew from the program (n=7) or failed to complete the intensive phase of the program within 70‐98 days (n=2). A total of 6 hospital admissions were recorded for 5 patient participants (10.2% of 49 program participants) within 30 days of the patients’ commencement of the VHHHFP (based on the patient’s recorded start or enrollment date). One patient was admitted twice in 3 days within the 30 days. The earliest admission within the 30 days occurred at 7.8 days, the latest at 27.9 days.

A further 8 hospital admissions for 7 patients (14.2% of participants) were recorded between days 31 and 98 of the patients’ commencement of the VHHHFP (Table 4). None of these patients had previously recorded an admission within 30 days. One patient was admitted twice in 3 days within the 31‐ to 98-day period. The earliest admission during this period occurred at 36.7 days, the latest at 89.9 days. In total, 14 separate hospital admissions were recorded during the intensive phase of the VHHHFP across 12 patients (24.5% of program participants). However, the data provided were not complete and consistent for all hospital admissions to determine the reason or cause for the admission (ie, all-cause, HR-related, or otherwise) for the 14 hospital admissions.

**Table 4.** Frequency of all causes and 30-day readmissions to the hospital during the intensive phase of the Virtual Home Health Heart Failure Program.

Frequency of hospital admissions	Number of patients, n (%)	Readmitted within 30 days of commencing the VHHHFP^a^, n (%)
No admissions	37 (75.5)	44 (89.8)
1	10 (20.4)	4 (8.2)
2	2 (4.1)	1 (2.0)

a VHHHFP: Virtual Home Health Heart Failure Program.

### Semistructured Interviews

A total of 9 patients participated in the interviews, 4 females and 5 males, ranging in age from 53 to 82 years. The VHHHFP was described by patients as a virtual resource that facilitated clinician interaction and care and was a platform for sharing clinical patient self-measures and communication. Three themes were identified from the analysis that described the patient experiences of the VHHHFP following completion of the 12-week intensive phase of the VHHHFP (Table S1 in Multimedia Appendix 2). The first theme described how the VHHHFP enhanced patient capability for self-management of HF. Patients described recognizing the need to engage in individualized self-care, and the program enhanced their ability and empowerment to confidently manage their HF. The second theme, improved patient comfort, was an outcome of engagement with the program, where patients described the VHHHFP as allaying patient fear and uncertainty regarding their HF condition, and the information and education (provided from the program) contributed to patient comfort and support from family. The third theme described positive influences on calm and how calm improved through coordinated care and a supportive environment. The virtual program contributed to this supportive environment.

A total of 6 health care professionals (4 nurses, 1 cardiologist, and 1 dietitian) shared their experiences and perceptions of the VHHHFP. For the study, the term “staff” will be used; however, in the VHHHFP, staff were either used directly by the organization delivering the service or as external providers. Through individual interviews, the experiences and perceptions of HCPs involved in the VHHHFP were explored (Table S2 in Multimedia Appendix 1). The first theme identified was health care professionals’ improving patient capability through a shared understanding of health needs. This theme included creating a supportive environment of care, the importance of guidelines for shared care, and health care professionals getting satisfaction from supporting patients in a virtual model of care. The second theme, improving capability through care practices, encompassed staff perception of making a difference to patient self-care as an outcome of the VHHHFP. This was described as achieved through the provision of care to maximize outcomes and patient capability and empowering patients in self-care practices. The third theme, promoting comfort and calm through virtual coordinated and collaborative care approach, described how staff identified that the VHHHFP contributed to patient comfort and calm. Recognition of the multidisciplinary model of care and that the virtual program enables partnership with the care team and patients was identified as critical components to the success of the program. Some participants also acknowledged that access to GPs or cardiologists presented a challenge sometimes. Table S2 in Multimedia Appendix 2 provides exemplars for the themes and subthemes.

## Discussion

### Principal Findings

This study contributes to the knowledge base on the impact of a virtual health approach from both a patient and clinician perspective and on how virtual health solutions can be integrated into existing care. A statistically significant improvement in physical health and well-being on completion of the intensive phase was noted as measured by the KCCQ-12. Drawing on previous research as a reference point [24], a 5-point threshold for meaningful clinical change has been proposed as equivalent to a ~10% relative reduction in the risk of adverse clinical events. Using this interpretation, a mean improvement of 21.7 in KCCCQ-12 patient summary scores from commencement to completion of the intensive phase of the VHHHFP could be considered to represent a 40% relative reduction in the risk of adverse clinical events pre- and post-intensive phase of the VHHHFP [24]. This highlights the beneficial effect of the program on patients’ physical health and well-being. The positive impact of postdischarge interventions on KCCQ-12 scores and, therefore, the well-being of patients with HF has also been demonstrated elsewhere. An intervention study by Stubblefield et al [31] comprising self-care activities, home visits, and telephone calls to coach participants in the aspects of HF self-management demonstrated a 5.4-point increase in KCCCQ-12 scores in the intervention group compared to the usual structured care group. The ongoing connection with clinicians following discharge from the hospital appears to contribute to the well-being of patients with HF. The quantitative findings reflect the qualitative themes of patients feeling “empowered to manage self-care activities,” that “information and education contributed to patient comfort,” and that the “virtual program provided a supportive environment.”

### Comparison to Prior Work

Anxiety and depression are known to be significant issues in patients with HF [30], impacting many areas of a patient’s life, including adherence to treatment plans. Anxiety and depression are also associated with reduced quality of life [32], reduced exercise capacity [33], and increased hospitalizations and mortality [34]. In this study, there was a statistically significant reduction in self-reported levels of anxiety and improvement in self-reported symptoms on the distress scale during the program, highlighting the importance of routinely monitoring mood in patients with HF. Enabling patients to self-report symptoms of anxiety and depression safely provides the opportunity for early recognition and management, which in turn may improve the management of HF and improve patient outcomes. This was reinforced in the qualitative data of this study, where patients reported feeling reassured when they had a clinician to contact if and when they had an exacerbation of their symptoms. Studies of patients with cardiac conditions have traditionally measured easily accessible outcomes such as hospitalizations and mortality. A strength of this study is the focus on patient-reported outcomes, which provides clinicians and researchers with an accurate report of health status directly from the patient, leading to the capture of meaningful data on the patient experience [35].

Enhanced patient capability is an important outcome for the program given the centrality of self-management in HF. This was highlighted in the qualitative results relating to patient experiences after engaging with the VHHHFP. The availability of staff to ask questions and allay concerns was described by patients as important and also contributing to their comfort, providing reassurance that someone was monitoring their measurements. This highlights the value of the availability of support beyond scheduled consultations. The CCC framework highlights the component of calm, which, from a patient’s perspective, reiterates the important benefits of having services designed around them rather than around the HCP, reducing the stress of accessing care and minimizing disruption to their lives. These qualitative findings could potentially be linked to the positive change in the PHQ-4 total distress score, which was statistically significant. Virtual programs such as the VHHHFP promote outcomes related to calm for postacute support services. The impact and importance of care integration have been evidenced in other studies and populations where continuity and availability of health care providers were recommended [36]. The findings from this study highlighted the need to ensure all HCPs involved in the patient’s care were familiar with the program’s services to ensure smooth communication processes. The impact of communication processes on calm has also been reported in other populations. In a study with people diagnosed with bipolar disorder, participants reported that their sense of calm was enhanced by increased engagement time and improved communication with health care providers [37], and in young adults diagnosed with cancer [38].

The qualitative data support a clinically significant improvement in self-reported knowledge, beliefs, confidence, and skills about managing HF throughout the intensive phase of the VHHHFP. Similar effects were reported in a study of patients with atrial fibrillation undertaking a virtual program during the COVID-19 pandemic. Improvements in self-monitoring abilities and self-management behaviors, and statistically significant reductions in anxiety and depression were also findings from this study [39]. Patients with HF are particularly vulnerable in the immediate post hospital discharge period while transitioning to their home environments.

The up-titration of guideline-directed medications is a cornerstone in patients with HF, particularly in the context of reduced ejection fraction [40], and is an essential strategy to fill the gap in care during the early discharge period [41]. Evidence-based clinical guidelines recommend that each medication be titrated as tolerated to the target dose, which was supported by landmark clinical trials to achieve maximum benefits [42]. Medication up-titration had limited uptake in this study despite processes for this being in place, including the availability of medication up-titration request forms. The qualitative data did not directly identify why the opportunity for up-titration was not readily taken up, but this could be related to the references to challenges in accessing GPs or cardiologists, where any changes to a titration plan required a GP or specialist oversight. The qualitative data indicate that nursing staff reported the need to titrate medications but did not have the protocol or scope to do so without contacting the patients’ GP or specialist. In a study exploring the barriers to up-titration of beta-blockers in patients with HF in the community, barriers identified included physicians’ concerns about medication side effects and polypharmacy, existing health care system barriers, comorbidities, patient communication, and physicians’ knowledge and experience [43]. The lack of uptake and potential missed opportunity to improve patient outcomes is an area for further exploration. An extended scope of practice for nurses to up-titrate and initiate guideline-directed medications could be an option for future iterations of the VHHHFP [44].

Data on hospital admissions were not collected from the time of discharge from the hospital, but from the time of enrollment into the program, which is a limitation of this study. However, the rate of hospital admissions within 30 days of enrollment into the VHHHFP was 10.2%, which does compare favorably to 30-day all-cause readmission rates in a previous study, which demonstrated 20% [45]. While a direct comparison cannot be made due to potential delays from discharge from the hospital to onboarding to the program, the results are still promising in that readmission was well below 20%. The finding is also in line with previous research that demonstrated a reduction in readmission rates in similar, virtually delivered, remote monitoring programs for patients with HF [14].

### Strengths and Limitations

A strength of this study was the exploration of self-reported patient outcomes and patient and clinical staff perceptions of the VHHHFP. The exploratory qualitative process evaluation provided valuable insights into the acceptability and usability of the intervention from the perspectives of the participants. The qualitative data allowed a deeper understanding of how participants responded to the program and the contextual factors that influenced the study outcomes. Trials in the study of patients with cardiac care routinely report outcomes such as hospitalizations and mortality. However, a significant strength of this study was the focus on patient-reported outcomes, an approach that could be more widely adopted in virtual health programs and beyond.

It is important to acknowledge the limitations that may affect the validity and generalizability of the research findings. First, the service was set up as a pilot to assess feasibility, and as such, the sample size was not powered to detect change with a level of statistical certainty. Second, the evaluation commenced after the commencement of the service, and as such, it was not possible to create a control group, minimizing the opportunity to assess selection or detection bias. The variation in the time taken to complete the intensive phase and the submission of data associated with key time points made comparisons between participants in the current evaluation challenging. To include as many participants as possible in the study, the parameters relating to the time taken to complete the intensive phase and the submission of data associated with a given time point were extended. Third, the number of patient participants who agreed to be interviewed postcompletion of the 12-week intensive program was low, and there may have been a self-selection bias among patients who chose to participate in the qualitative component of this evaluation. In addition, direct comparisons with data on readmission rates could not be made where the date of discharge from the hospital (the admission that led to the initial referral to the program) was not collected.

### Future Directions

The growth in virtual health programs, such as VHHHFP, has demonstrated a range of benefits to patients regarding improved access to advice and guidance on their medical condition without the need to visit health care facilities. This approach has led to the reallocation of much-needed health care resource provision [46], especially within the high-resource area of HF management. The virtual nature of the intervention creates the opportunity to scale within and across health care services. However, this service sits in what can be termed the “missing middle” of health care, with the service spanning a gap between care provided in the acute care and community settings. One of the key barriers to further implementation in the Australian context relates to funding mechanisms. Ongoing support for services that do not meet existing health care funding mechanisms can be uncertain, limiting their increased uptake and opportunity for further translation of benefits to a broader section of the community. Further research into enhancing the adoption of such models is needed. The use of technologies to support the management of HF is growing internationally. Understanding how virtual health care that uses remote patient monitoring can be integrated into existing systems and models of care is a challenge that requires multilevel collaboration. The findings of this study support the need to develop and adopt virtual health care solutions for chronic disease management, including and beyond HF.

### Conclusion

The evaluation of the VHHHFP demonstrates improvements in both clinical and behavioral outcomes, directly addressing our primary study aim. Patients completing the program showed statistically significant improvements in all KCCQ-12 domains, including physical limitations, quality of life, symptom frequency, and social limitations. Psychological health measures similarly improved with reductions in total distress and anxiety scores. Our second aim, exploring participant experiences, revealed enhanced capability, improved comfort, and positive influences on calm according to the VBHC framework. Clinician experiences identified benefits in patient capability through shared decision-making and care practices, while also noting virtual coordination promoted patient comfort and calm. This evaluation, using both quantitative and qualitative methods, provides evidence for the effectiveness of the VHHHFP model in HF management.

## Supplementary material

10.2196/64877 Multimedia Appendix 1 Open-ended participant interview questions.

10.2196/64877 Multimedia Appendix 2 Themes and exemplar quotes generated from patient and health care professional interviews.

## Abbreviations

CCC: Capability, Comfort, and Calm
CNS: clinical nurse specialist
GP: general practitioner
HCP: health care practitioner
HF: heart failure
KCCQ-12: Kansas City Cardiomyopathy Questionnaire-12
LACE: Length of stay, Acuity of admission, Comorbidity, and Emergency room usage
PAM-13: Patient Activation Measure-13
PHQ-4: Patient Health Questionnaire-4
PREM: Patient Reported Experience Measure
VBHC: value-based health care
VHHHFP: Virtual Home Health Heart Failure Program
