# Supplementary material for: Evaluation of a Virtual Home Health Heart Failure Program: Mixed Methods Study
Source: JMIR Cardio. 2025 Jul 23;9:e64877. doi: 10.2196/64877 (PMC12309783; doi:10.2196/64877)
Supplement: Multimedia Appendix 1 [file cardio-v9-e64877-s001.docx]

Appendix 1

Participant Interview Questions

1. Introduction to self
   1. First name
   2. Age
   3. How would you describe in a few words, what living with heart failure is like?
2. Diagnosis:
   1. Tell us about the first time you were told you had heart failure? How did you feel at that moment? What did you do next?
   2. Tell us about your other health problems.
3. Daily management of disease: *(capability)*
   1. What do you have to do to take care of yourself in terms of your heart failure?

*Prompt – so your Biometric monitoring - measuring of vital signs regularly*

- 1. How do you cope in day-to-day life with self-management of heart failure?
  2. Do you sometimes feel that it is hard to do all these things to take care of your health?
  3. Do you ever skip any of the things you should do?
  4. What helps when it comes to living with this condition? OR what support really works for you?

1. Emotions: *(comfort)*
   1. For some, the personal work they have to do to in relation to their disease is emotionally challenging. Is this something you can relate to?
   2. Is there something you do to keep your spirits up?
2. Relationship with health professionals: *(calm)*
   1. Could you tell me about your relationship with different health professionals?

*Prompt -so for example using the health care system, so the link with the hospital, as well as the various other staff such as the nurses, physio, dietitian, nursing*

*Prompt – so for example having someone to help coordinate your care, someone you have regular contact with*

- 1. How did you find the communication between your GP, cardiologist and the VHH team?
  2. How has the additional support from the VHH team assisted you in the management of your heart failure?

*Prompt – for example when you feel unwell, SOB dizziness, etc, using your heart failure action plan*

*Prompt – perhaps you could tell me about a time when you were given advice or education from the Team that made a difference to how you were feeling.*

*Prompt – so would you say this education was valuable or made the most difference to you.*

1. Social support: *(calm)*
   1. What is the role of your family, friends, or other caregivers in relation to your heart failure?
   2. In relation to your heart failure, do you get any help from others?
   3. What types of assistance do you think would help with the management of your heart failure? (Family or healthcare social support)
2. Conclusion
   1. Is there anything you want to talk about that we didn’t mention today?

*Prompt – so to summarise can you share your overall experience with the virtual home heart program.*

Healthcare Team Member Interview Questions

1. What was your overall experience with the virtual home hospital for heart failure patients?

*Prompt – for example how easy was it to integrate the service into your regular duties?*

1. Outside of this program who/what else does the clinician regularly consult/refer to in relation to patients with heart failure? (cardiologist, HF nurses, dietitian, pharmacists).

*Prompt – for example your experience of identifying and referring patients to the service?*

1. Do clinicians feel they are well trained in heart failure management, or do they feel they gave gaps in their knowledge?

*Prompt – for example an example of escalation*

1. How comfortable do you feel about prescribing heart failure guideline directed medications and up titrating to goal doses? (Ask GPs and cardiologist) (ask HF Nurse about following a protocol for this same question)
2. Where do clinicians see their scope of responsibilities or roles in relation to HF patient? How do they see the roles of others? e.g., GPs manage day to day difficulties and cardiologists manage heart function.

*Prompt – would this link to communication*

- 1. Do you feel that your perception on these responsibilities have changed since having the VVH service to assist?

*Prompt – for example do you see the value of the service*

1. Do clinicians feel certain patient characteristics or attitudes influence how they manage their care? i.e., living alone, medication adherence or frail
2. Can you think of a recent unplanned heart failure readmission and describe circumstances involved, who were the key agents, and did they think it could have been avoided and how?
3. Conclusion:
   1. Is there anything you want to talk about that we didn’t mention today?

*Prompt – such as suggestions for improvements to the VHH HF service?*
