# Supplementary material for: Evaluation of a Virtual Home Health Heart Failure Program: Mixed Methods Study
Source: JMIR Cardio. 2025 Jul 23;9:e64877. doi: 10.2196/64877 (PMC12309783; doi:10.2196/64877)
Supplement: Multimedia Appendix 2 [file cardio-v9-e64877-s002.docx]

**Table S1**. Themes and associated exemplars generated from patient interviews.

| Theme | Subtheme | Exemplars |
| --- | --- | --- |
| Theme 1: enhanced patient capability | | |
|  | Improving capability through patient development skills | - *I counted every bit of fluid I put into myself, not quite so strict now, but because I counted everything for a long time, are now know exactly of how much I can have.* [P1] - *The skills that they've given me, the routine that they've given me, are fantastic. I feel great, compared to what I was.* [P3] |
|  | Empowered to manage self-care activities. | - *They're all practical. It keeps me on my toes with what I've got to do at home and to make sure that I'm meeting the goals, you know, which are set.* [P9] |
| Theme 2: improved patient comfort | | |
|  | Allaying patient fear  and uncertainty. | - *When I was in the hospital and diagnosed with heart failure. I just thought, crikey, you know what's going to happen now when I get home, and I have to look after myself. So that thought was quite daunting.* [P1] - *When they said you've got heart failure, I looked at him and I thought, well, you know, what does that mean? It's I'm still alive. My heart hasn't failed.* [P3] |
|  | Information and education contributed to patient comfort. | - *Yeah, in the beginning, when I was having weekly consults with xx. He was always trying to educate me on what I had to do, what I could expect, and all that sort of thing. Yeah, telling me all about it. Yeah, it's very good.* [P1] - *And they're great to talk to. Any questions, not a problem. I mean, they've been fantastic. Brilliant.* [P3] |
|  | Support from family. | - *She's well aware of the whole program and supports it.* [P9] - *My wife did support. …Make sure I do the blood pressure in the morning with my measuring of vital signs, and regularly weigh. Very proactive and personable. Help me. You know she's great.* [P2] |
| Theme 3: positive influences on calm | | |
|  | Calm improved through coordinated care. | - *And I think having the virtual nurse, you can ask whatever you like, cause it's you and him, or you and her. So, anything that's troubling you is bothering you.* [P7] - *Talking to him and then explaining to me why they do it, why you're doing this when they're speaking to him once a week, then once a fortnight now, once a month, there's been fantastic. It's answered a lot of questions. Yeah, and that's great. I would recommend it to anybody who's had this problem.* [P3] |
|  | Virtual program provided a supportive environment. | - *And I think it's very. Very appropriate. That was the word I was going to respond to. It is appropriate and it is manageable, and I think it's well directed. It's focused on. And I thought I knew it all, but I obviously didn't.* [P4] - *I think it sort of gives you a bit more confidence about what you're doing as well, especially when you don't have the hospital around the corner like I have so.* [P5] |

**Table S2**. Themes and exemplars generated from health care professional interviews.

| Theme | Subtheme | Exemplars |
| --- | --- | --- |
| Theme 1: improving patient capability through a shared understanding of health needs | | |
|  | Creating a supportive environment of care | - *You do build up that little bit more of a rapport with them. And a bit of that connection.* [P2] - *You know, you build with the patient that relationship that is, I think, one of the main reasons why, as well, that we’re able to have such a successful program.* [P1] |
|  | Importance of guidelines for shared care | - *I’m still getting skills in and confidence, but there is a good protocol to follow. And I've always got xxx to talk to if I need to.* [P4] - *So, the protocols in place are for standard for everyone, which guides us to do what we need to do, but at the end of the day, we also correlate them with the patients’ symptoms and that's how we're able to do things and that's what the protocol dictates as well.* [P1] |
|  | Satisfaction from supporting patients. | - *I am very. Happy with, you know my experience, and hopefully the patients do feel the same way as well.* [P1] - *I think it's a great program and I've been loving it. I've always been a bedside nurse. But doing this virtually, …that's a different sort of nursing that I've never done before, which is interesting and rewarding.* [P3] |
| Theme 2: improving capability through care practices | | |
|  | Provision of care to maximise outcomes and patient capability | - *We can prevent, you know, patients from falling ill.* [P1] - *I think it's amazing. I think it was something that is lacking and something a service that is usually extremely vital to all tertiary cardiology care...But with xxx hospitals being a unique service, this has certainly filled the gap and has, in my point of view, decreased morbidity and hospitalizations for all our heart failure patients.* [P6] |
|  | Empowering patients in self-care practices | - *Do these things with the patients where I can guide them and make sure to reduce their risk of readmission, and give them the skills to look after themselves much better moving forward as well.* [P1] - *Some are super compliant, and you know, adhere to their restrictions or diet and exercise regimen.* [P4] |
| Theme 3: promoting comfort and calm through a virtual coordinated and collaborative care approach | | |
|  | Recognition of a multidisciplinary model of care | - *There's a lot on it. So, you're going to stay on your toes, but if there is an issue, you've got it to refer to.* [P3] - *I think they've (GP’s) been excellent. That they feel like they've been very involved, I hope. The team has tried their best to involve the GPs within the whole management team.* [P6] |
|  | Virtual program enables partnership with the care team and patients | - *…patients can access, you know, expert nursing and allied healthcare, and also specialist care as well, from the fact that we can be that link between their treating specialists and them in wherever they are around Australia.* [P2] - *Close communication with the cardiologist or whoever is the referring specialist allows me to guide the patient.* [P1] |
